# Assessment of quality of life, pain level and disability outcomes after lumbar discectomy

**DOI:** 10.1038/s41598-023-33267-z

**Published:** 2023-04-12

**Authors:** Rafał Staszkiewicz, Uladzislau Ulasavets, Paweł Dobosz, Szymon Drewniak, Ewa Niewiadomska, Beniamin Oskar Grabarek

**Affiliations:** 1 Department of Neurosurgery, 5th Military Clinical Hospital with the SP ZOZ Polyclinic in Krakow, 30-901 Krakow, Poland; 2 Department of Histology, Cytophysiology and Embryology, Faculty of Medicine in Zabrze, Academy of Silesia, 4-055 Katowice, Poland; 3 Department of Laryngology, 5th Military Clinical Hospital with the SP ZOZ Polyclinic in Krakow, 30-901 Kraków, Poland; 4 Department of Anesthetics, 5th Military Clinical Hospital with the SP ZOZ Polyclinic in Krakow, 30-901 Kraków, Poland; 5 Department of Epidemiology and Biostatistics, School Health Sciences in Bytom, Medical University of Silesia, 41-902 Bytom, Poland; 6 Laboratory of Molecular Biology and Virology, 40-851 Katowice, Poland

**Keywords:** Public health, Quality of life, Spondyloarthritis

## Abstract

This study aimed to assess the quality of life of 113 Caucasian patients with intervertebral disc (IVD) degeneration of the lumbosacral (L/S) spine who qualified for microdiscectomy during a 12-month period after surgery. Based on magnetic resonance imaging before the surgery, the degree of radiological advancement of the degenerative changes was determined according to the Pfirrmann grading scale from 1 to 5. To assess pain intensity, the Visual Analog Scale (VAS) was used; the Satisfaction with Life Scale (SWLS) was used to evaluate quality of life; and to assess the degree of ability, the Oswestry Low Back Pain Disability Questionnaire (ODI) was employed. The level of pain, assessed using the VAS, significantly changed in the months following the surgery, with the highest values noted before surgery and the lowest a year after. In turn, the results of the SWLS questionnaire revealed a significant increase in satisfaction with life in the subsequent stages of the study. The conducted correlation analysis revealed significant dependencies in terms of quality of life in regard to pain as well as degree of disability. The level of pain and degree of disability were closely related to the degree of radiological advancement of degenerative changes according to the Pfirrmann grading scale.

## Introduction

Intervertebral disc (IVD) degeneration is a significant and potentiating therapeutic as well as social problem^1^. IVD degeneration is treated conservatively using pain-relieving and anti-inflammatory medication, most often nonsteroidal anti-inflammatory drugs (NSAIDs), as well as rehabilitation. Surgical treatment is used when previous conservative treatment does not lead to an improvement or when degenerative changes are extensive enough that surgery is the treatment of choice^2–4^.

The occurrence of IVD degeneration of the lumbosacral (L/S) spine section is predisposed by several factors: significant reduction in physical activity; remaining in a position that is unfavorable for the spine for long periods of time, including sitting; microtrauma that damages the intervertebral disc; obesity; heavy lifting; and old age. Genetic predisposition is also an important factor, such as family history of the disease. Environmental factors, including smoking, diabetes, vascular diseases, infections, chronic stress, depression, type of professional work and poor physical fitness, also increase the risk of back pain^5,6^.

The predominant symptom of IVD degeneration is pain in a specific section of the spine, which is estimated to occur in 70–90% of some populations over 40 years of age^5,6^. Undoubtedly, symptoms associated with IVD degeneration significantly reduce quality of life^7^.

Chronic and recurrent spine pain can affect up to 60% of the population and constitutes one of the most common reasons for contacting family doctors in our country. However, the fact that respondents often attribute pain in the back region to an affliction of the spine, regardless of a correct diagnosis or identification of the true cause, should be borne in mind^5^.

At times, when disease symptoms are significant enough that they prevent the leading of a so-called “normal life”, patients must change or resign from their current work and thus transfer to disability pension^8,9^. Furthermore, the patient’s social life suffers, as at times, patients are forced to limit their social life in addition to engaging in long-term medication (mainly pain medication) use. Due to pain, patients may resign from their current work and hobbies, remaining in bed until symptoms subside^10^.

Moreover, it should be borne in mind that progressive IVD degeneration limits movement capacity. Due to the chronicity of symptoms and the period of exacerbation, patients are discouraged from being physically active, ultimately leading to a reduction in quality of life^11^.

The issue of quality of life (standard of living, lifestyle) has been present among people since the existence of civilization. In 1994, the World Health Organization (WHO) defined quality of life as “an individual’s perception of their position in life in the context of the culture and value systems in which they live and in relation to their goals, expectations, standards and concerns”^12^. This means that quality of life depends on the person, on their hierarchy of values and needs by which they lead themselves and which are important for them. Based on this definition, it can be determined that quality of life has different meanings for everyone, and different elements and situations will influence its assessment. Quality of life is a subjective value, and according to the WHO, it depends on physical state, mental state, social relations, environment, religion, beliefs, convictions, and views^13,14^. The Satisfaction with Life Scale (SWLS) is a questionnaire used in the self-assessment of life satisfaction, relating to mental health, subjective quality of life, and the likelihood of suicide attempts^15^. Since 1995, tools for the assessment of health-related quality of life have been used in ill individuals experiencing lumbosacral spine pain as well as sciatica and in patients undergoing surgery due to a prolapsed disc in the lumbosacral spine. A specific tool for the assessment of pain in the L/S segment is the Oswestry Disability Index (ODI)^16,17^.

The aim of this prospective study was to assess the quality of life and disability of patients with IVD degeneration of the L/S spine section with regard to the radiological advancement of degenerative changes who qualified for microdiscectomy in the 12-month period following the procedure.

## Results

In all patients, pain continued for at least 1 year; moreover, it was observed that the occurrence of advanced degenerative changes was significantly related to the period of time that pain persisted, more frequent receiving of NSAIDs and the ability to work and partake in sports (Table 1). The significantly highest body mass was noted among patients with degree 5 advancement of changes (p < 0.05).

**Table 1:** Characteristics of the patients qualified for microdiscectomy due to degeneration of the intervertebral disc of the L/S spine section.

Characteristics	Total N = 113 (100)	Degree using the Pfirrmann grading scale				p value
		2 n = 27 (23.9)	3 n = 43 (38.1)	4 n = 32 (28.3)	5 n = 11 (9.7)	
Duration of chronic back pain (years)						
1–3	14	8 (57.1)	1 (7.1)	5 (35.7)	0 (0)	0.006
4–6	30	7 (23.3)	14 (46.7)	8 (26.7)	1 (3.3)	
7–10	29	9 (31)	10 (34.5)	5 (17.2)	5 (17.2)	
> 10	40	3 (7.5)	18 (45)	14 (35)	5 (12.5)	
Gender						
Female	55	12 (21.8)	19 (34.6)	19 (34.6)	5 (9.1)	0.56
Male	58	15 (25.9)	24 (41.4)	13 (22.4)	6 (10.3)	
Body weight (kg)	113	64.2 ± 3.0	69.1 ± 4.5	64.0 ± 2.9	71.1 ± 4.9	< 0.0001
BMI (kg/m^2^)	113	27.2 ± 1.4	28.8 ± 2.4	22.0 ± 2.2	30.1 ± 1.0	< 0.0001
Education						
Elementary/Junior high school	7	2 (28.6)	0 (0)	3 (42.9)	2 (28.6)	0.14
Vocational	59	10 (16.9)	28 (47.5)	17 (28.8)	4 (6.8)	
Secondary	39	12 (30.8)	11 (28.2)	11 (28.2)	5 (12.8)	
Higher	8	3 (37.5)	4 (50)	1 (12.5)	0 (0)	
Professional situation						
Employed	93	25 (26.9)	40 (43)	27 (29)	1 (1.1)	< 0.0001
Not working	20	2 (10.0)	3 (15.0)	4 (20.0)	11 (55.0)	
Smoking						
No	77	18 (23.4)	31 (40.3)	22 (28.6)	6 (7.8)	0.88
Yes	36	9 (25)	12 (33.3)	11 (30.6)	4 (11.1)	
Drinking alcohol						
No	8	4 (50)	2 (25)	1 (12.5)	1 (12.5)	0.30
Yes	105	23 (22.0)	41 (39.0)	31 (29.5)	10 (9.5)	
The frequency of alcohol consumption						
Never	8	4 (50)	2 (25)	1 (12.5)	1 (12.5)	0.17
Once a month	35	7 (20)	11 (31.4)	12 (34.3)	5 (14.3)	
2–4 times a month	61	16 (26.2)	23 (37.7)	18 (29.5)	4 (6.6)	
≥ 2 times a week	9	0 (0)	7 (77.8)	1 (11.1)	1 (11.1)	
Sports						
No	103	21 (20.4)	40 (38.8)	31 (30.1)	11 (10.7)	0.04
Yes	10	6 (60.0)	3 (30.0)	1 (10.0)	0 (0)	
How many hours a day at work/university/home do you spend sitting?						
1–2	32	13 (40.6)	10 (31.3)	8 (25)	1 (3.1)	0.008
3–5	44	10 (22.7)	11 (25)	15 (34.1)	8 (18.2)	
6–9	24	3 (12.5)	12 (50)	7 (29.2)	2 (8.3)	
> 10	13	1 (7.7)	10 (76.9)	2 (15.4)	0 (0)	
The frequency of taking nonsteroidal anti-inflammatory drugs per week						
0	4	4 (100)	0 (0)	0 (0)	0 (0)	< 0.0001
1	15	7 (46.7)	5 (33.3)	3 (20)	0 (0)	
2	35	18 (51.4)	12 (34.3)	5 (14.3)	0 (0)	
3	31	2 (6.5)	18 (58.1)	11 (35.5)	0 (0)	
4	9	0 (0)	4 (44.4)	5 (55.6)	0 (0)	
5	6	0 (0)	3 (50)	2 (33.3)	1 (16.7)	
6	2	0 (0)	0 (0)	1 (50)	1 (50)	
7	11	(0)	1 (9.1)	1 (9.1)	9 (81.8)	

Qualitative data are presented as numbers and percentages; p values were determined by the chi-squared test. Quantitative data are presented as the mean ± standard deviation; p values by ANOVA.

Significant diversity was not determined for the advancement of changes under the influence of smoking cigarettes, drinking alcohol, or education in the groups (p > 0.05).

### Relationship between VAS, ODI and SWLS scores

The level of pain felt was assessed using the VAS scale and significantly changed in the months following the procedure, with the highest values being noted before the operation, while the lowest were noted a year after the procedure. There were no statistically significant differences between men and women. The average assessment of pain at the moment the study started was 6 on a scale from 1 to 10. This assessment was taken into account later as a point of reference for the division of results using the VAS scale. The number of obtained points on the ODI scale was considered to indicate a downward trend, even in the case of patients with the highest degree of radiological advancement of degenerative changes (degree 5), according to the Pfirrmann scale. Furthermore, the results of the SWLS questionnaire reveal a significant increase in satisfaction with life in the subsequent stages of the study. Bearing in mind specific stages of the study, significant differences in the assessment of pain, disability, and satisfaction were observed in specific groups based on the Pfirrmann scale (Table 2).

**Table 2:** Average assessment of pain intensity according to the VAS scale, degree of disability according to the ODI scale, and quality of life according to the SWLS scale of patients qualified for the microdiscectomy procedure, overall and divided for each radiological degree of advancement of degenerative changes according to the Pfirrmann scale.

Scale	Moment (month)	Total N = 113 (100)	Gender		p value	Degree using the Pfirrmann grading scale				p value	p < 0.05
			Female	Male		2 n = 27 (23.9)	3 n = 43 (38.1)	4 n = 32 (28.3)	5 n = 11 (9.7)		
VAS (points)	0	6 (5–8)	6 (6–8)	6 (4–8)	0.18	4 (3–4)	6 (6–6)	8 (8–8)	10 (9–10)	p < 0.0001	2 vs. 3, 4, 5 3 vs. 4, 5
	3	6 (5–8)	6 (5–8)	5 (4–8)	0.31	4 (4–4)	6 (5–6)	8 (6–8)	9 (8–10)	p < 0.0001	2 vs. 3, 4, 5 3 vs. 4, 5
	6	3 (3–5)	4 (3–5)	3 (3–5)	0.11	2 (2–3)	3 (3–3)	5 (4–5)	8 (7–10)	p < 0.0001	2 vs. 3, 4, 5 3 vs. 4, 5
	12	3 (2–3)	3 (2–4)	2 (2–3)	0.06	2 (2–2)	2 (2–3)	4 (3–4)	8 (6–9)	p < 0.0001	2 vs. 4, 5 3 vs. 4, 5
	p value	p < 0.0001	p < 0.0001	p < 0.0001	–	p < 0.0001	p < 0.0001	p < 0.0001	p < 0.0001	–	
ODI (points)	0	18 (13–27)	21 (13–29)	16 (13–26)	0.21	12 (7–13)	16 (14–19)	29 (26–31)	30 (25–32)	p < 0.0001	2 vs. 3, 4, 5 3 vs. 4, 5
	3	16 (11–23)	18 (10–23)	15 (11–23)	0.54	9 (5–11)	15 (12–18)	26 (22–29)	28 (21–31)	p < 0.0001	2 vs. 3, 4, 5 3 vs. 4, 5
	6	15 (7–21)	16 (6–21)	15 (7–21)	0.71	4 (1–6)	15 (11–16)	21 (18–25)	23 (19–30)	p < 0.0001	2 vs. 3, 4, 5 3 vs. 4, 5
	12	12 (3–20)	15 (2–20)	11 (4–19)	0.57	0 (0–2)	11 (9–16)	21 (18–25)	22 (15–28)	p < 0.0001	2 vs. 3, 4, 5 3 vs. 4, 5
	p value	p < 0.0001	p < 0.0001	p < 0.0001	–	p < 0.0001	p < 0.0001	p < 0.0001	p < 0.0001	–	
ODI (%)	0	36 (26–54)	42 (26–58)	32 (26–52)	0.21	24 (14–26)	32 (28–38)	58 (52–62)	60 (50–64)	p < 0.0001	2 vs. 3, 4, 5 3 vs. 4, 5
	3	32 (22–46)	36 (20–46)	30 (22–46)	0.71	18 (10–22)	30 (24–36)	52 (44–58)	56 (42–62)	p < 0.0001	2 vs. 3, 4, 5 3 vs. 4, 5
	6	30 (14–42)	32 (12–42)	29 (14–42)	0.56	8 (2–12)	30 (22–32)	42 (36–50)	46 (38–60)	p < 0.0001	2 vs. 3, 4, 5 3 vs. 4, 5
	12	24 (6–40)	30 (4–40)	22 (8–38)	0.54	0 (0–4)	22 (18–32)	41 (35–49)	44 (30–56)	p < 0.0001	2 vs. 3, 4, 5 3 vs. 4, 5
	p value	p < 0.0001	p < 0.0001	p < 0.0001	-	p < 0.0001	p < 0.0001	p < 0.0001	p < 0.0001	–	
SWLS (points)	0	20 (15–24)	19 (10–23)	21 (18–24)	0.05	27 (24–30)	21 (19–22)	16 (9–19)	7 (5–11)	p < 0.0001	2 vs. 3, 4, 5 3 vs. 4, 5
	3	21 (17–24)	21 (13–24)	21 (17–25)	0.34	27 (24–30)	21 (18–23)	18 (13–20)	11 (7–13)	p < 0.0001	2 vs. 3, 4, 5 3 vs. 5
	6	22 (19–27)	22 (18–25)	23 (19–27)	0.13	27 (24–31)	22 (20–26)	21 (18–23)	13 (11–18)	p < 0.0001	2 vs. 3, 4, 5 3 vs. 5
	12	24 (21–28)	24 (20–28)	25 (22–29)	0.35	28 (26–31)	25 (22–27)	22 (20–25)	19 (16–22)	p < 0.0001	2 vs. 4, 5
	p value	p < 0.0001	p < 0.0001	p < 0.0001	-	p = .81542	p < 0.0001	p < 0.0001	p < 0.0001	–	

Quantitative data presented as median (1st quartile- 3rd quartile); p values by the Mann‒Whitney U test and the nonparametric ANOVA tests: the Friedman test for repeated measures, The Kruskal‒Wallis test.

### Correlation of VAS, ODI and SWLS scale assessments

The conducted correlation analysis revealed significant dependencies in the assessment of quality of life in regard to pain and degree of disability (Table 3). A higher level of satisfaction was correlated with a lower level of pain as well as a higher level of fitness.

**Table 3:** The correlation strength of pain intensity according to the VAS scale, degree of disability according to the ODI scale, and quality of life according to the SWLS scale of patients qualified for the microdiscectomy procedure, overall and divided for each radiological degree of advancement of degenerative changes according to the Pfirrmann scale.

Scale	Moment (month)	Total N = 113 (100)	Degree using the Pfirrmann grading scale			
			2 n = 27 (23.9)	3 n = 43 (38.1)	4 n = 32 (28.3)	5 n = 11 (9.7)
SWLS (pts) vs VAS (pts)	0	− 0.77; p < 0.0001	− 0.18; p = 0.38	− 0.17; p = 0.26	0.04; p = 0.81	− 0.27; p = 0.42
	3	− 0.71; p < 0.0001	− 0.25; p = 0.2	− 0.05; p = 0.76	− 0.14; p = 0.44	− 0.5; p = 0.12
	6	− 0.59; p < 0.0001	− 0.04; p = 0.86	− 0.28; p = 0.07	− 0.07; p = 0.69	− 0.52; p = 0.1
	12	− 0.42; p < 0.0001	–	− 0.02; p = 0.92	0.1; p = 0.57	− 0.64; p = 0.03
SWLS (pts) vs ODI (pts)/ODI (%)	0	− 0.68; p < 0.0001	0.46; p = 0.01	− 0.07; p = 0.65	− 0.33; p = 0.07	0.002; p = 0.99
	3	− 0.63; p < 0.0001	0.46; p = 0.02	0.01; p = 0.95	− 0.26; p = 0.15	− 0.55; p = 0.08
	6	− 0.6; p < 0.0001	0.27; p = 0.18	− 0.44; p = 0.003	− 0.08; p = 0.66	− 0.11; p = 0.76
	12	− 0.47; p < 0.0001	0.21; p = 0.29	− 0.36; p = 0.02	− 0.07; p = 0.7	0.43; p = 0.19

Data are presented as Spearman’s rank coefficient of correlation; p value using Spearman’s rank correlation test.

The level of pain and the degree of disability were closely related to the degree of radiological advancement of degenerative changes according to the Pfirrmann scale (Table 4). Improvement in quality of life was observed significantly more often among people with a lower degree of advancement of the disease.

**Table 4:** Point-based assessment of pain intensity according to the VAS scale, degree of disability according to the ODI scale and quality of life according to the SWLS scale of patients qualified for the microdiscectomy procedure, overall and divided for each radiological degree of advancement of degenerative changes according to the Pfirrmann scale.

Moment (month)	Scale		Total n = 113 (100)	Degree using the Pfirrmann grading scale				p value
				2 n = 27 (100)	3 n = 43 (100)	4 n = 32 (100)	5 n = 11 (100)	
VAS								
0		Below 6	34 (30.1)	27 (100)	7 (16.3)	0 (0)	0 (0)	< 0.0001
		6 and more	79 (69.9)	0 (0)	36 (83.7)	32 (100)	11 (100)	
3		Below 6	49 (43.4)	27 (100)	19 (44.2)	3 (9.4)	0 (0)	< 0.0001
		6 and more	64 (56.6)	0 (0)	24 (55.8)	29 (90.6)	11 (100)	
6		Below 6	96 (85)	27 (100)	42 (97.7)	27 (84.4)	0 (0)	< 0.0001
		6 and more	17 (15)	0 (0)	1 (2.3)	5 (15.6)	11 (100)	
12		Below 6	98 (86.7)	27 (100)	43 (100)	28 (87.5)	0 (0)	< 0.0001
		6 and more	15 (13.3)	0 (0)	0 (0)	4 (12.5)	11 (100)	
ODI (pts)								
0		None	2 (1.8)	2 (7.4)	0 (0)	0 (0)	0 (0)	< 0.0001
		Slight	34 (30.1)	21 (77.8)	13 (30.2)	0 (0)	0 (0)	
		Moderate	41 (36.3)	4 (14.8)	29 (67.4)	6 (18.8)	2 (18.2)	
		Severe	34 (30.1)	0 (0)	1 (2.3)	25 (78.1)	8 (72.7)	
		Immobile/bed-bound	2 (1.8)	0 (0)	0 (0)	1 (3.1)	1 (9.1)	
3		None	4 (3.5)	3 (11.1)	1 (2.3)	0 (0)	0 (0)	< 0.0001
		Slight	42 (37.2)	24 (88.9)	18 (41.9)	0 (0)	0 (0)	
		Moderate	41 (36.3)	0 (0)	24 (55.8)	14 (43.8)	3 (27.3)	
		Severe	26 (23)	0 (0)	0 (0)	18 (56.3)	8 (72.7)	
		Immobile/bed-bound	0 (0)	0 (0)	0 (0)	0 (0)	0 (0)	
6		None	18 (15.9)	16 (59.3)	2 (4.7)	0 (0)	0 (0)	< 0.0001
		Slight	33 (29.2)	11 (40.7)	19 (44.2)	3 (9.4)	0 (0)	
		Moderate	48 (42.5)	0 (0)	22 (51.2)	20 (62.5)	6 (54.5)	
		Severe	13 (11.5)	0 (0)	0 (0)	8 (25)	5 (45.5)	
		Immobile/bed-bound	1 (0.9)	0 (0)	0 (0)	1 (3.1)	0 (0)	
12		None	31 (27.4)	26 (96.3)	5 (11.6)	0 (0)	0 (0)	< 0.0001
		Slight	32 (28.3)	1 (3.7)	26 (60.5)	3 (9.4)	2 (18.2)	
		Moderate	39 (34.5)	0 (0)	12 (27.9)	21 (65.6)	6 (54.5)	
		Severe	10 (8.8)	0 (0)	0 (0)	7 (21.9)	3 (27.3)	
		Immobile/bed-bound	1 (0.9)	0 (0)	0 (0)	1 (3.1)	0 (0)	
ODI (%)								
0		None	2 (1.8)	2 (7.4)	0 (0)	0 (0)	0 (0)	< 0.0001
		Minimal	11 (9.7)	8 (29.6)	3 (7)	0 (0)	0 (0)	
		Moderate	49 (43.4)	17 (63)	32 (74.4)	0 (0)	0 (0)	
		Severe	34 (30.1)	0 (0)	8 (18.6)	20 (62.5)	6 (54.5)	
		Immobile	17 (15)	0 (0)	0 (0)	12 (37.5)	5 (45.5)	
3		None	4 (3.5)	3 (11.1)	1 (2.3)	0 (0)	0 (0)	< 0.0001
		Minimal	23 (20.4)	17 (63)	6 (14)	0 (0)	0 (0)	
		Moderate	43 (38.1)	7 (25.9)	30 (69.8)	4 (12.5)	2 (18.2)	
		Severe	35 (31)	0 (0)	6 (14)	23 (71.9)	6 (54.5)	
		Immobile	8 (7.1)	0 (0)	0 (0)	5 (15.6)	3 (27.3)	
6		None	18 (15.9)	16 (59.3)	2 (4.7)	0 (0)	0 (0)	< 0.0001
		Minimal	18 (15.9)	11 (40.7)	7 (16.3)	0 (0)	0 (0)	
		Moderate	47 (41.6)	0 (0)	30 (69.8)	14 (43.8)	3 (27.3)	
		Severe	26 (23)	0 (0)	4 (9.3)	16 (50)	6 (54.5)	
		Immobile	4 (3.5)	0 (0)	0 (0)	2 (6.3)	2 (18.2)	
12		None	31 (27.4)	26 (96.3)	5 (11.6)	0 (0)	0 (0)	< 0.0001
		Minimal	11 (9.7)	1 (3.7)	10 (23.3)	0 (0)	0 (0)	
		Moderate	47 (41.6)	0 (0)	27 (62.8)	16 (50)	4 (36.4)	
		Severe	22 (19.5)	0 (0)	1 (2.3)	14 (43.8)	7 (63.6)	
		Immobile	2 (1.8)	0 (0)	0 (0)	2 (6.3)	0 (0)	
SWLS								
0		Decrease in quality of life	52 (46)	0 (0)	16 (37.2)	25 (78.1)	11 (100)	< 0.0001
		State unchanged	6 (5.3)	0 (0)	5 (11.6)	1 (3.1)	0 (0)	
		Increase in quality of life	55 (48.7)	27 (100)	22 (51.2)	6 (18.8)	0 (0)	
3		Decrease in quality of life	49 (43.4)	0 (0)	17 (39.5)	22 (68.8)	10 (90.9)	< 0.0001
		State unchanged	3 (2.7)	0 (0)	0 (0)	3 (9.4)	0 (0)	
		Increase in quality of life	61 (54)	27 (100)	26 (60.5)	7 (21.9)	1 (9.1)	
6		Decrease in quality of life	34 (30.1)	0 (0)	10 (23.3)	15 (46.9)	9 (81.8)	< 0.0001
		State unchanged	4 (3.5)	1 (3.7)	2 (4.7)	1 (3.1)	0 (0)	
		Increase in quality of life	75 (66.4)	26 (96.3)	31 (72.1)	16 (50)	2 (18.2)	
12		Decrease in quality of life	18 (15.9)	1 (3.7)	3 (7)	8 (25)	6 (54.5)	0.0004
		State unchanged	6 (5.3)	0 (0)	2 (4.7)	4 (12.5)	0 (0)	
		Increase in quality of life	89 (78.8)	26 (96.3)	38 (88.4)	20 (62.5)	5 (45.5)	

Qualitative data are presented as numbers and percentages; p values were determined by the chi-squared test.

**Figure 1:**
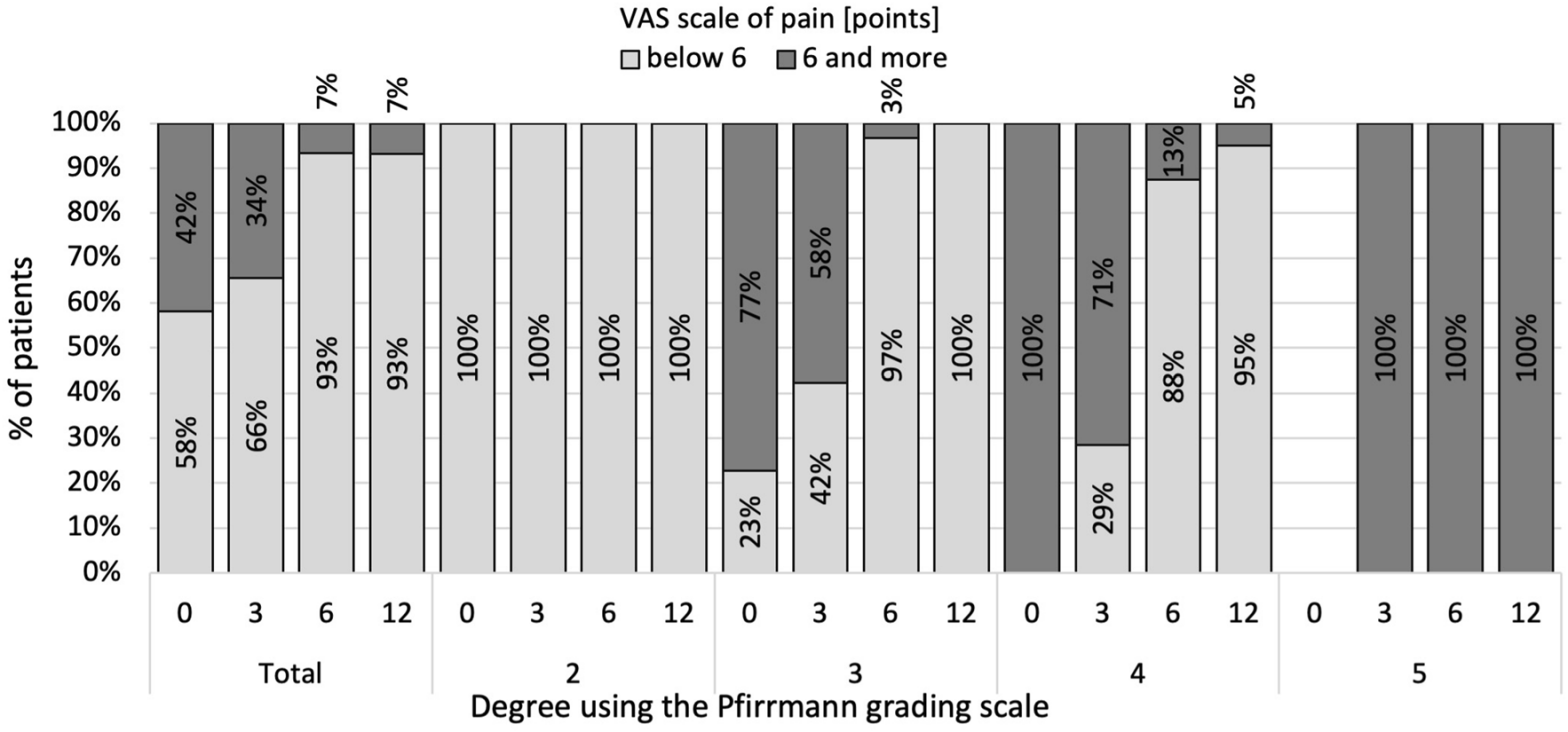
Percentage of patients in whom there was an improvement in quality of life in groups due to the radiological degree of advancement of degenerative changes according to the Pfirrmann scale, accounting for the pain level.

Among all the respondents, the percentage of patients in whom there was an improvement in quality of life increased in the months following the operation in the group of people who classified their pain below 6 on the VAS scale (Fig. 1).

The initial data showed that the chance of satisfaction with life significantly decreased with an increased assessment of pain above a score of 6 (OR 0.03; 95% CI 0.006–0.116). Similar results were observed after 3 months—OR 0.83; 95% CI 0.045–0.268, 6 months—OR = 0.15; 95% CI: 0.049–0.482, and 12 months—OR 0.12; 95% CI 0.037–0.388.

**Figure 2:**
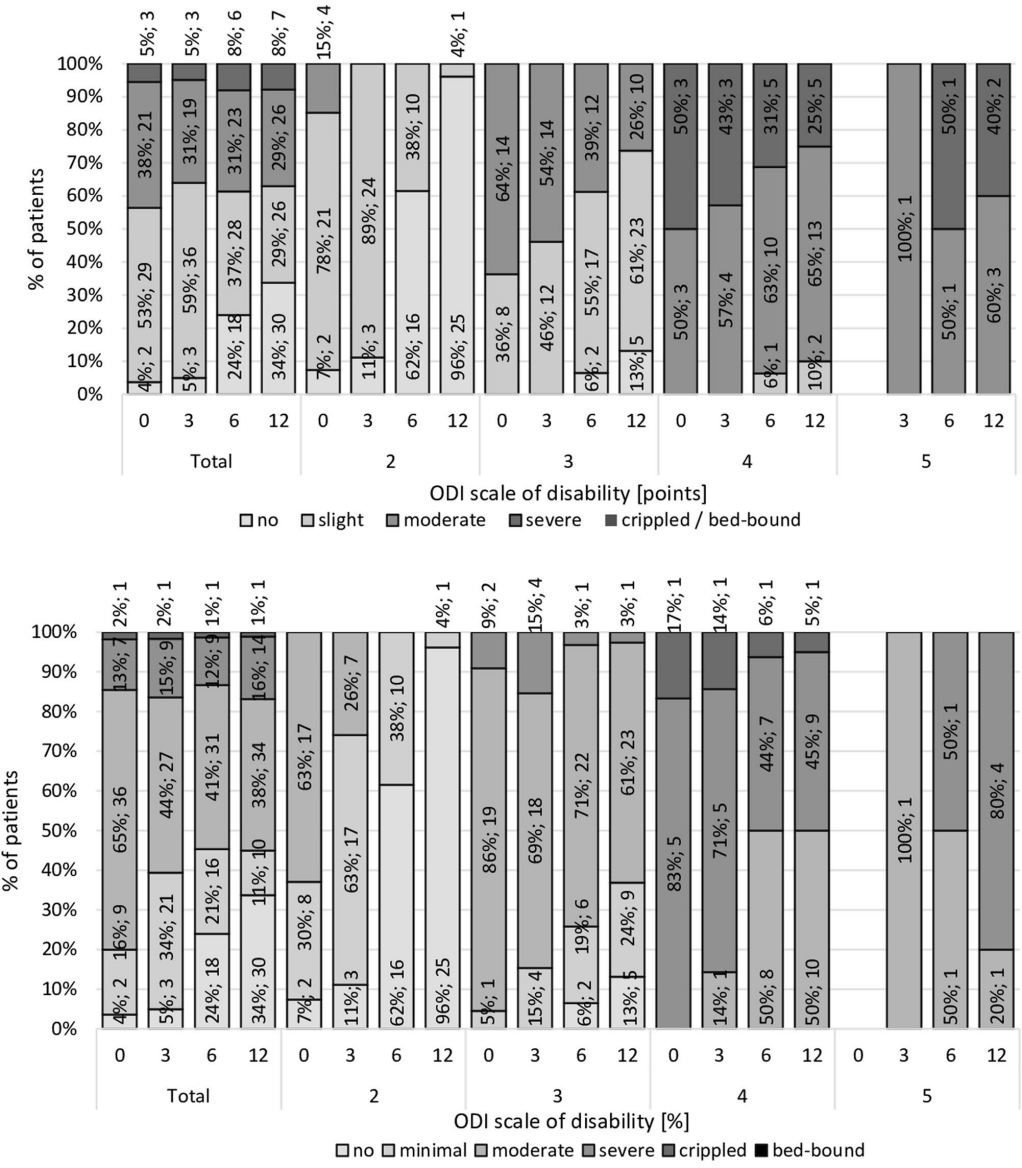
Percentage of patients in whom there was an improvement in quality of life in groups due to the radiological degree of advancement of degenerative changes according to the Pfirrmann scale, accounting for the level of disability according to (**B**) the 5- and (**A**) 6-point ODI scale.

In the months following the surgery, alongside an improvement in the quality of life, increasing participation from patients with higher fitness was also visible (Fig. 2). The initial data revealed that the chance of satisfaction with life significantly decreased with a 1-point increase in the result of the SWLS questionnaire (OR 0.79; 95% CI 0.735–0.862). Similar results were observed after 3 months—OR 0.83; 95% CI 0.778–0.892, 6 months—OR 0.86; 95% CI 0.809–0.921, and 12 months—OR 0.91; 95% CI 0.860–0.964.

## Discussion

There have been numerous RCTs and systematic reviews conducted with the same research purposes and even larger numbers of patients; however, the vast majority of these studies have concentrated on assessing quality of life, IVD degeneration, and patient disability before surgery and at specific postoperative periods, without considering the degree of radiological advancement of degenerative changes, according to the Pfirrmann scale, as a criterion.

When we conducted the assessment of available literature using the PubMed database, using the key words “quality of life” AND “Pfirrmann scale” OR “radiological advancement of degenerative changes”, the total number of articles from 1977 to 2022 was 226.

Using the generally accepted exclusion criteria, the number of articles in English totaled 202, of which there were 189 full-text articles available, and when the criterion of human studies was applied, 143 articles were initially qualified. Next, we (Rafał Staszkiewicz and Beniamin Oskar Grabarek) independently analyzed these 143 articles, finding that only 5 were directly related to our study objective^18–22^. It is also notable that none of these articles involved the Polish population. We followed the same procedure when searching the database using the words “disability” AND “Pfirrmann scale” OR “radiological advancement of degenerative changes”, which allowed us to initially indicate 287 articles, of which 256 were in English, 242 were available as full-text articles and 190 regarded humans. However, this time, none of these articles touched on the target topic.

Therefore, we believe that the analysis we conducted is very significant and constitutes an important supplementary study in terms of quality of life and disability (Health Science) among patients with degeneration of the L/S spine section.

In our study, we assessed quality of life among patients whose degree of radiological advancement of degenerative L/S spinal changes and pain led to qualification for neurosurgical microdiscectomy of the L/S spine.

The first part of the analysis we conducted confirmed that an increase in the advancement of IVD degenerative changes in the L/S spine section was accompanied by a statistically significant increase in the frequency of taking NSAIDs. Significantly, almost all patients (81.8%) with degree 5 degenerative changes took pain medication every day. This indicates that the patients' daily lives were marked by considerable pain and suffering.

However, we did not find that habitual consumption, such as alcohol consumption or cigarette smoking, has an influence on the occurrence of IVD degeneration of the L/S segment. This is in line with the observations of Zhang et al., who critically reviewed the literature and reported that moderate alcohol consumption is a factor preventing IVD degeneration^23^. In turn, smoking cigarettes is an unfavorable prognostic factor for IVD degeneration, as confirmed by Chen et al.^24^.

We then assessed pain intensity, quality of life, and degree of disability among patients, both before the neurosurgical procedure and in the 12-month period following it. First, we noted that the greater the degree of IVD degeneration, the more patients reported pain of a higher intensity. It should be noted that patients with degree 5 degenerative changes declared maximum pain intensity (10 points), which decreased by only 2 points 12 months after the operation.

In most patients, pain was a daily occurrence. This pain was of moderate or strong intensity in nature. These studies confirmed that unfortunately, pain is an inherent element of degenerative spine disease and affects the lives of both patients and their families^25^. Research by Chin et al. among patients with atypical changes and low back pain confirmed a statistically significant improvement in terms of pain and level of disability after microdiscectomy. Patients, both with and without steatosis and edema of the spinal bone marrow, showed a significant improvement in the postoperative VAS score for low back pain^26^.

Our results correspond with the data of other authors who studied pain intensity in relation to IVD disease using the VAS scale. It should be noted that both the observation period and data collection vary, which makes comparing results more difficult. Puolakka et al. analyzed risk factors for absence from work caused by L/S spine section pain in patients after surgery. The observation period in these patients was 5 years after surgery, while 2 months after the operation, data on pain intensity were collected based on the VAS scale. A significant reduction in pain following the procedure was reported^27^.

In turn, Solberg et al. conducted a study that aimed to assess pain intensity among patients with IVD degeneration of the L/S spine section before a neurosurgical procedure and 12 months after. In the case of pain in the L/S spine region, the pain intensity decreased from 5.17 to 2.13 (p < 0.001), whereas in the case of appendage pain, it decreased from 6.34 to 1.68 (p < 0.001). In both assessments, the reduction in pain was significant and noticeable^28^. It follows that regardless of the observation period, the surgical procedures performed due to IVD degeneration of the L/S section result in a noticeable reduction in pain.

Furthermore, using a dedicated ODI questionnaire for the assessment of the degree of disability caused by degenerative changes in the L/S spine section, we determined that overall, microdiscectomy resulted in a decrease in disability, assessed at the beginning from 18 points (moderate disability) to 12 points (minimal disability). None of the patients declared full disability; however, the more intense the radiological changes were, the lower the fitness of the patients was.

The observations described by Middendrop et al. confirmed that the degree of radiological advancement of IVD degenerative changes of the L/S spine section positively correlates with decreased fitness in patients. These authors found that the ODI results of the patients fluctuated, ranging from 0 to 91.11% (arithmetic mean 32.77% ± 17.02%). The largest group of patients (48.39%) had moderate functional disability (ODI result between 21 and 40%)^29^.

Additionally, Hasanović-Vučković et al. assessed the fitness of 100 patients with degenerative disease of the L/S segment, noting a distribution close to ours for the number of patients at individual degrees of radiological advancement of degenerative changes, according to the Pfirrmann scale (1–0.75%, 2–14.25%, 3–37.5%, 4–36.5%, and 5–11%)^22^. In our study, the largest number of patients had degree 3 (38.1%) and degree 4 (28.3%) IVD degenerative changes.

These authors reported a statistically significant correlation between the degree of advancement of changes and VAS (r = 0.16) and ODI (r = 0.24) scores^22^. In turn, Gautschi et al. assessed the relationship between the degree of radiological advancement of degenerative changes and postoperative pain intensity, impairment of functionality, and health-related quality of life (HRQoL) among 336 patients. These authors did not indicate a direct relationship between radiological advancement of radiological changes and clinical evaluation^30^. Additionally, Corniola et al. did not confirm a relationship between the radiological advancement of changes in the L/S spine in patients qualified for surgical treatment and the scores on commonly used pain scales. However, in patients with degree 4 and 5 radiological changes, there was a clear tendency for a higher pain intensity and a reduction in quality of life^18^.

Significantly, from the viewpoint of the importance of our research, Mazza et al. demonstrated the effectiveness of conservative treatment among patients with degree 3 and 4 advancement of degenerative changes of the L/S segment, according to the Pfirrmann scale, in a group of only 23 patients. They determined a reduction in pain during the 24-week observation^20^.

Nonetheless, Grasso et al. confirmed effectiveness, reduction in pain, and improvement in quality of life among patients in whom hybrid surgery was conducted due to radiculopathy or cervical myelopathy^21^.

We also indicated that the best results for the surgery were achieved at the lowest degrees of advancement of degenerative changes. Furthermore, we confirmed a strong correlation between the level of pain, disability, and quality of life. A higher level of satisfaction was correlated with a lower level of pain as well as a higher level of fitness. Improvement in the quality of life was significantly more often observed among people with a lower degree of disease advancement, although larger differences in quality of life between the subsequent observation months were noted for higher degrees of disease advancement. The percentage of patients with improved quality of life increased in the months following the operation in the group who classified their pain below 6 on the VAS scale. It follows that pain plays a large role in how well-being is perceived by patients, and its reduction or elimination is a particularly important issue.

In the case of the initial data, the chance of being satisfied with life significantly decreased with a 1-point increase in the result for the SWLS questionnaire.

Kurowska et al. proved that people who have diseases of the lower segment of the spine and have developed depression experience chronic pain of a much greater intensity^31^.

Other authors also noted a significant improvement in the quality of life and life satisfaction after surgery due to IVD degeneration of the L/S spine section-sectional area, cross-sectional area difference index (CDI) and fat infiltration rate (FIR) of the multifidus, erector spinae and psoas major at the apical vertebral level were measured using MRI. The visual analogue scale (VAS) score, Oswestry Disability Index (ODI) and 36-item Short Form Health Survey (SF-36) score were used to evaluate patient quality of life. Correlations between the degree of asymmetric muscular degeneration and quality of life were analysed. The CDI of the multifidus, erector spinal and psoas major was higher in the DLS group compared with that in the control group. The CDI of the multifidus was found to be positively associated with the Cobb angle of lumbar scoliosis. Similar results were obtained for fat infiltration between the two groups. In addition, the CDI and FIR difference index of the multifidus was positively correlated with the VAS score and ODI but negatively correlated with the SF-36 score. The quality of life significantly decreased with increasing asymmetric atrophy and fat infiltration in the multifidus. Thus, strategies to enhance the function of the multifidus may have a positive impact on quality of life^32,33^. Of course, it should be remembered that the microdiscectomy procedure alone did not have an influence on the improvement in quality of life; it is likely that the rehabilitative treatment after surgery, coordinated by a rehabilitation specialist, also played a role.

Our study has both strengths and weaknesses. A strength of this study is that the analysis was conducted on a relatively large number of respondents who underwent microdiscectomy in one clinical center, considering that different surgical methods are also used for treating IVD degeneration of the L/S segment. Additionally, pain intensity, degree of fitness, satisfaction with life, and the success of neurosurgical treatment based on the degree of advancement of degenerative changes in the IVD of the L/S spine section according to the Pfirrmann scale were also evaluated. In the available literature, there are few observations on the quality of life among patients with IVD degeneration of the L/S spine section depending on the degree of radiological advancement of lesions^18–22^. The first limitation of our study is the observation period of 12 months after microdiscectomy. Of course, it would be very interesting and cognitively important to assess the quality of life in this group of patients over a period longer than 1 year. The second limitation is the fact that this was a single-center study, in which the number of patients representing the individual degrees of IVD degeneration was not uniform. Although the total number of patients may seem small, our statistical analysis indicated that 113 cases constituted a sufficiently representative group, allowing for analysis to be conducted and a reliable result to be obtained. Furthermore, pain and its influence on everyday functioning is a subjective feeling that is dependent on many factors. Its perception is influenced by emotional, pathological, genetic, and other cognitive elements. Of course, the obtained results might also have been influenced by the fact that we used only one questionnaire that is commonly employed to assess quality of life. However, using a questionnaire consisting of a larger number of questions, such as the SF-36, would allow us to assess quality of life with consideration of other criteria. Nevertheless, when comparing the strengths and weaknesses, conducting our study can be determined as justified, as it contains valuable research findings. It is a milestone study in research regarding quality of life and disability in patients following a microdiscectomy procedure due to IVD degeneration of the L/S spine.

In summary, the pain level, which was assessed using the VAS scale, significantly changed in the months following the surgery, with the highest values noted before the surgery and the lowest a year after the operation. The results of the SWLS questionnaire revealed a significant increase in satisfaction with life in the subsequent stages of the study. When considering individual stages of the study, significant differences in the assessment of pain, disability, and satisfaction were observed in individual groups based on the Pfirrmann grading scale. MRI is a significant component in the diagnosis of degenerative disease, while microdiscectomy is an effective surgical method for the treatment of degenerative changes in the IVDs of the L/S spine. Furthermore, our analysis indicated that the highest chance of returning to fitness, at a level equal to or close to the level prior to the occurrence of IVD degeneration, after the microdiscectomy procedure occurs in patients at early degrees of radiological advancement, such as 2 and 3. Therefore, patients should not delay the decision to undergo surgery when there are clinical grounds for it. The role of the neurosurgeon is to explain to the patient in detail the essence of the disease, possible treatments, advantages, and possible side effects of surgery. Therefore, the physician must partner with the patient and exhibit highly developed communication skills.

## Materials and methods

### Participants

In this study, 113 Caucasian patients, consisting of 55 women (48.7%) and 58 men (51.3%) who were qualified for surgical treatment of intervertebral disc degeneration of the lumbosacral spine (microdiscectomy) were enrolled. The surgeries were carried out in the period from March 2020 to April 2021, always by the same team of neurosurgeons, under the leadership of Rafał Staszkiewicz. The observation period ended in May 2022. All patients were treated and underwent surgery in the same clinic, Department of Neurosurgery, 5th Military Clinical Hospital with the SP ZOZ Polyclinic in Krakow, 30-901 Krakow, Poland. The inclusion and exclusion criteria were the same as in our previous paper^34^ and are presented in Table 5.

**Table 5:** Inclusion and exclusion criteria.

Inclusion criteria	Exclusion criteria
Over 18 years of age	Under 18 years of age
Isolated lumbosacral spine interverbal disc with degeneration of a prolapse or extrusion character determined through MRI	Degeneration of the IVDs of the lumbosacral spine of a protrusion or sequestration character determined through MRI
Discogenic pain and/or symptomatic sciatica accompanied by back pain, gluteal pain, or leg pain without improvement after nonoperative treatment for at least 6 weeks	Previous surgical procedures due to degeneration of the IVDs of the lumbosacral spine
No other coexisting pathologies of the spine	Inflammatory and autoimmune diseases
Exacerbation of pain (acute pain) < 12 weeks absence of neurological loss symptoms	Condition after spine injury
	Dementia/mental disorders
	Polyneuropathy
	Pregnancy
	Coexisting diseases, including metabolic diseases
	Neoplasms: metastatic tumor in the spine; lymphoma; leukemia; spinal cord tumors; retroperitoneal tumors; primary shaft tumors
	Inflammatory diseases: inflammation of the bone elements of the spine
	Osteoporosis
	Duration of disease exceeding 12 weeks and no shorter than 6 weeks
	Presence of neurological impairment symptoms such as foot drop, cauda equina symptoms, perineal sensory disturbances, impaired urinary and bowel control

Each patient underwent a neurological examination including an assessment of muscle strength; passive movement; muscle tension; patellar, ankle, plantar, Babinski sign, and Rossolimo sign reflexes; surface and deep sensation; gait; mobility; and L/S spine pain. The study excluded cases of patients who presented with loss symptoms, as such patients required emergency neurosurgical intervention; we undertook evaluation only among stable cases (Table 5). All patients included in the study declared pain in the buttocks and radiating pain to the left lower limb—56 patients (49.56%); right—54 patients (47.78%) or both—3 patients (2.66%).

**Figure 3:**
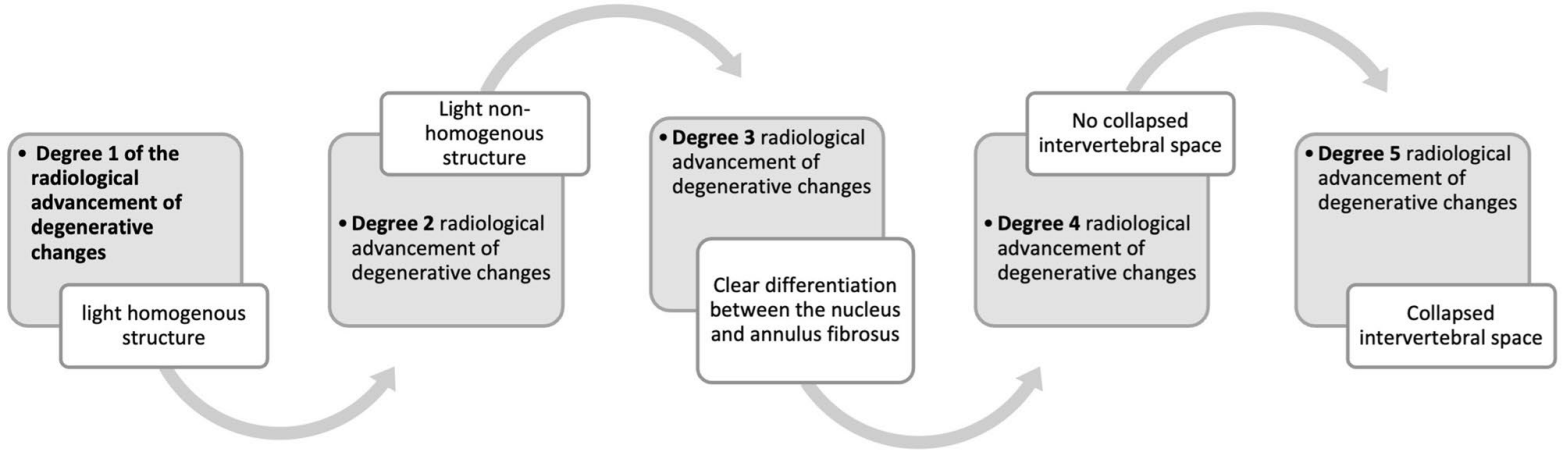
Protocol for the assessment of the radiological advancement of degenerative changes in human IVDs using the Pfirrmann scale [own drawing].

In all patients, before the microdiscectomy procedure, a magnetic resonance imaging examination was conducted (Signa Hde 1.5 T General Electric Medical System, Poland) using the projections SE T1, SE T1 FLuid-Attenuated Inversion Recovery (FLAIR), FSE T2 and Short Tau Inversion Recovery (STIR) sequences in transverse and sagittal sections in 3 mm and 4 mm thick layers. This allowed for the determination of the degree of radiological advancement of degenerative changes, according to the Pfirrmann scale from 1 to 5 (Fig. 3). The analysis of the MRI images, including the determination of the degree of IVD degeneration according to the Pfirrmann scale, was performed independently by two neurosurgeons (Rafal Staszkiewicz, MD, PhD, and Dorian Gładysz, MD) employed at the Department of Neurosurgery, 5th Military Clinical Hospital with the SP ZOZ Polyclinic in Krakow, 30-901 Krakow, Poland. In a situation where the abovementioned specialists determined a different degree of lesions, the Department Head—Wiesław Strohm, MD, PhD—had the decisive opinion. Out of 113 cases, only in three cases (2.65%) did the assessment of the degree of IVD degenerative changes differ.

In 27 (23.9%) patients, radiological changes were classified as grade 2 degeneration; in 43 (38.1%) patients, they were classified as grade 3; in 32 (28.3%) patients, they were classified as grade 4; and 11 (9.7%) patients were classified as having grade 5 advancement of changes on the Pfirrmann scale. Furthermore, qualification for surgery consisted of conducting a neurological examination as well as conducting physical and subjective examinations in each patient.

After being discharged from the Department of Neurosurgery, patients were required to report themselves to the Neurosurgery Clinic within 4 weeks of the procedure for routine inspection. Subsequently, patients were recommended rehabilitation and were informed about subsequent proceedings.

Patients were asked to complete all questionnaires before surgery (month 0) as well as 3, 6 and 12 months after it was carried out.

### Microdiscectomy procedure

In each patient, the microdiscectomy procedure was performed under general anesthesia in the abdominal position. A 2–4 cm skin incision was made over the affected interbody space, identified intraoperatively by fluoroscopic X-ray. Once the right space was identified and the skin incision was made, the posterior surface of the vertebral arches was exposed with the help of special surgical instrumentation, holding the previously separated spinal muscles. This was followed by an incision of the yellow ligament, which was lifted in a small space, opening the lumen of the spinal canal. After assessing the intracanal conditions and exposing the hernia, the IVD and IVD sequestrator were removed. After the procedure was completed, a drain was placed at the wound site to drain accumulated blood, which was then removed after 24 h. Patients were verticalized on the first postoperative day, and early rehabilitation in the Neurosurgery Department was included. The patient's length of stay, including preoperative diagnosis, anesthesia consultation and early postoperative period, was 5 days.

### The satisfaction with life scale

The respondents answered 5 questions, which were measured using a 7-point scale, wherein 1 on the scale signified “definitely disagree” with the statement, while 7 signified “definitely agree” with the statement. The test has high internal consistency and test–retest correlations.

In our study, the SWLS questionnaire on quality of life was assessed for 3 possible situations:

1. Unchanged state (20 points)
2. Decrease in quality of life (1–19 points)
3. Increase in quality of life (21–25 points)

### Assessment of the pain level

**Figure 4:**
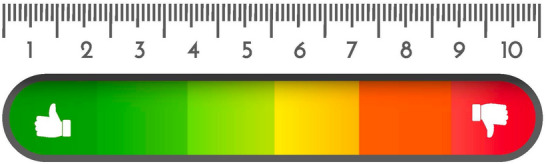
Visual Analog Scale—a special (colored) ruler for pain assessment [own drawing].

Pain intensity was assessed in all patients using the Visual Analog Scale (VAS) in the form of a line, on which the examined individual indicated their pain intensity on a scale from 0 to 10, where 0 signified no pain and 10 signified intense pain^30,35–38^ (Fig. 4).

### Assessment of the ODI value based on the Oswestry questionnaire

The patient answered 10 questions regarding (1) pain intensity; (2) self-handling (washing, dressing themselves, etc.); (3) picking up items; (4) walking; (5) sitting; (6) standing; (7) sleeping; (8) sex life (if applicable); (9) social life; (10) travel. The result is presented on a point scale (0–50 points, where 0 indicates a lack of dysfunction, and 50 indicates the worst condition of the patient) or on a percentage scale.

Using the 5-point scale, an assessment of the degree of disability was performed as shown below^39^:

0–4 points—lack of disability;

5–14 points—little disability;

15–24 points—moderate disability;

25–34 points—serious disability;

Above 35 points—complete disability.

Using the 6-point percentage scale, ODI factors were determined and interpreted as follows:

0–10%—no disability;

10–20%—minimal disability;

21–40%—moderate disability;

41–60%—severe disability;

61–80%—immobility;

81–100%—bed-bound patients.

### Statistical analysis

Analysis of the data was conducted using the procedures of the Statistica 13.3 (StatSoft, Poland) program. Categorical variables were presented using numbers and percentages of cases in each group. The χ^2^ independence and Fisher tests were used. Numeric data were presented as the mean with a standard deviation (X ± SD) in the case of a normal distribution; otherwise, they were presented as a median with quartiles—M (Q1–Q3). Compliance with a normal distribution was verified using the Shapiro‒Wilk test. The significance of differences in groups was checked using the Mann‒Whitney U test, the Kruskal‒Wallis test and the Friedman test for repeatable measurements. Correlation analysis was conducted using Spearman’s correlation coefficient with a significance test. Additionally, odds ratios (ORs) with 95% confidence intervals (95% CIs) were calculated based on univariate logistic regression models. A p value below 0.05 was considered statistically significant.

### Sample size calculation

According to data published by the Central Statistical Office (GSO) in 2019, the number of inhabitants of Poland was 38,383,000. The number of participants in the study was determined using the statistical tool available at https://www.naukowiec.org/dobor.html (accessed on 21 March 2020)^40^. For this population, the maximum error value was estimated at 9%. Therefore, assuming a p value < 0.05, the required number of respondents in the study was 119 (p value < 0.05). In turn, according to data obtained from the European Health Survey in 2019, 25.8% of adult Poles above 18 years old (n = 31,435,677) suffered from pain in the L/S section of the spine^41^. For this population, the maximum error value was estimated at 9%, and the required number of participants in the study was 100 (p value < 0.05).

### Ethical approval

This study was performed in accordance with the guidelines of the 2013 Declaration of Helsinki on human experimentation. Approval of the Bioethical Committee operating at the Medical University of Silesia, no KNW/022/KB/42/15 24 February 2015 and at the Regional Medical Chamber in Krakow, No. 162/KBL/OIL/2021 11 June 2021, was obtained for this study.

### Informed consent

Written informed consent was obtained from all patients.

## Data Availability

All data generated or analyzed during this study are included in this published article.
